# A feasibility study on a machine-learning-based quality assurance tool for spot-scanning proton therapy using delivery log files and treatment plans^☆^

**DOI:** 10.1016/j.phro.2026.100987

**Published:** 2026-04-30

**Authors:** Sang Kyun Yoo, Sridhar Yaddanapudi, Bo Lu, Ethan Stolen, Siddhant Sen, Byongsu Choi, Jin Sung Kim, Keith Furutani, Chris Beltran, James J. Sohn

**Affiliations:** a Yonsei Institute for Digital Health, Yonsei University, Seoul 03722, the Republic of Korea; b Department of Radiation Oncology, Yonsei Cancer Center, Heavy Ion Therapy Research Institute, Yonsei University College of Medicine, Seoul 03722, the Republic of Korea; c Department of Radiation Oncology, Mayo Clinic, Jacksonville, FL 32224, United States; d Department of Radiation and Cellular Oncology, University of Chicago, Chicago, IL 60637, United States; e Department of Psychology, University of Illinois Urbana-Champaign, Champaign, IL, United States

**Keywords:** Spot-scanning proton therapy, Machine learning, Spot position prediction, Plan accuracy, Quality assurance

## Abstract

**Background and purpose:**

Although pencil beam scanning provides superior dose conformity, delivery uncertainties remain in beam monitoring and steering systems. This study evaluates a machine learning model that predicts delivered spot positions from plan parameters as a complementary quality assurance support tool.

**Materials and methods:**

The dataset consisted of a single routine quality assurance treatment plan and 64 corresponding delivery log files from a Hitachi proton scanning beam system (32 files each from gantry 1 and 2), collected over three months across various proton energies. Statistical analyses, including Levene’s test, Welch’s analysis of variance, and Games-Howell post hoc tests, assessed the effects of beam energy, treatment day, and room on spot position errors. Machine learning regression models were trained using 48 delivery log files to predict delivered spot positions from treatment planning data. Model performance was assessed using mean squared errors, R^2^ score, and Euclidean distance.

**Results:**

Machine learning models demonstrated feasibility of spot position prediction with high accuracy (R^2^ = 0.999; mean squared errors of 0.021 mm^2^ and 0.003 mm^2^), with Euclidean errors <0.13 mm. Predicted dose distributions closely matched planned distributions, with an average absolute mean dose difference of 2.34 × 10^−5^ Gy, and a maximum absolute voxel-wise dose difference of 1.796 Gy, defined as the largest absolute difference between predicted and planned dose values at corresponding voxels.

**Conclusions:**

This feasibility study demonstrates that machine-learning-based prediction of delivered spot positions can achieve sub-millimeter accuracy, potentially enhancing the precision and reliability of quality assurance processes in proton therapy.

## Highlights

• A machine learning model was developed for QA in proton therapy.
• Delivered and planned spot positions were compared using log files and DICOM plans.
• The model achieved an R^2^ of 0.999 for both x- and y-coordinate predictions.
• Our framework supports proton therapy QA by predicting spot delivery positions.

## Graphical abstract

**Figure:**
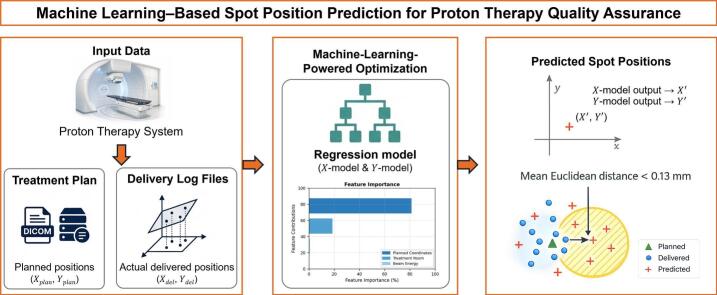

## 1 Introduction

Pencil beam scanning (PBS) has become the clinical standard in proton therapy due to its superior dose conformity [1], [2], [3]. The American Association of Physicists in Medicine (AAPM) Task Group (TG) 224 report emphasizes the critical importance of precision in proton therapy delivery, recommending specific tolerances for monitor units (MU) delivery and spot position [4]. Even small deviations can lead to clinically relevant dosimetric differences, particularly in regions with steep dose gradients or when systematic errors accumulate across multiple fractions [5], [6].

While several studies have investigated differences between planned and delivered beam parameters [7], [8], most have focused on measurement-based verification or retrospective analysis of delivery data. Commercial software tools and various in-house solutions have demonstrated high accuracy and reproducibility in automated, log-file-based quality assurance (QA) [9], [10]. For systems such as the PROBEAT-V (Hitachi, Ltd., Tokyo, Japan), efficient and comprehensive QA remains challenging because delivery log files are stored in a proprietary binary format that is not directly compatible with existing commercial QA tools and requires additional decoding and preprocessing. Previous studies have demonstrated log-file–based QA for the PROBEAT-V system, achieving high accuracy in reproducing delivered dose distributions and verifying delivery accuracy [11], [12]. However, the retrospective nature of these workflows provides limited support for identifying spot delivery deviations before subsequent treatment fractions.

Machine learning (ML) and data-driven approaches have become increasingly important in radiation therapy, enabling improvements in treatment planning, outcome prediction, and QA [13], [14], [15]. In contrast to prior reconstruction-based methods, the present study introduces an ML-based framework that learns from log and plan data to predict spot delivery deviations, providing a data-driven approach that complements conventional QA by identifying potential delivery deviations before clinical application.

Comparing planned parameters stored in Digital Imaging and Communications in Medicine (DICOM) plan files with delivered parameters recorded in log files can be automated and has been effectively implemented in many existing QA workflows. However, these approaches remain largely focused on retrospective verification of delivery accuracy. In this context, our feasibility study demonstrates that ML-based prediction can achieve clinically relevant accuracy, providing a complementary foundation for future validation and clinical implementation.

## 2 Materials and methods

### 2.1 Data description

In this study, we analyzed delivery log files, known as demand data, obtained from repeated deliveries of a single routine QA treatment plan on a synchrotron-based proton therapy system (Hitachi PROBEAT-V) [16]. The demand data contains spot positions, width and dose, as recorded by the spot position monitor (SPM) and dose monitor. The PROBEAT-V delivery log files are stored in a binary format and consist of spot-wise records, from which relevant beam parameters were extracted and synchronized with the planned spot information for analysis.

**Fig. 1:**
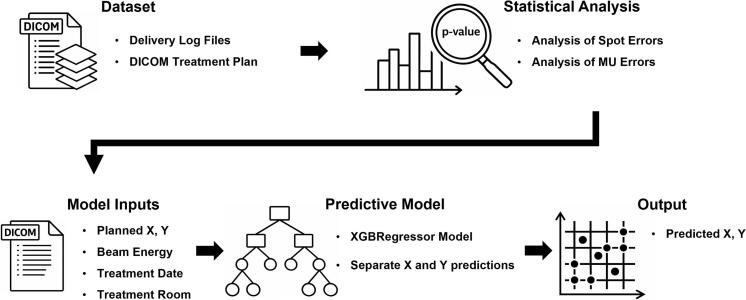
Overview of machine-learning-powered tool for quality assurance in proton therapy.

The overall workflow of the proposed QA framework is summarized in Fig. 1. The dataset used in this study consisted of a single treatment plan for routine QA, exported from the treatment planning system in DICOM ion plan format, together with corresponding delivery log files recorded by the proton delivery system. The same plan was repeatedly delivered during QA sessions over a three-month period at a fixed gantry angle, yielding a total of 64 delivery log files, with 32 files each from gantry 1 and 2 treatment rooms (G1 and G2). Log files from G1 and G2 were collected on matched QA-session dates (paired room-day sampling). This QA plan followed a spot-based PBS pattern across 20 energy layers at a fixed gantry angle, rather than a uniform cubic target-dose prescription. With a fixed gantry angle, a total of 233.3 MU was delivered according to the treatment plan, utilizing 20 proton energies (99.2 MeV–124.8 MeV) and a total of 928,000 proton spots across all 64 delivery log files.

The SPM monitors the position of each spot in real-time, and the information is recorded in the delivery log files. The planned spot positions were obtained from the DICOM ion treatment plans and were defined at the isocenter plane. To compare the delivered spot positions with the planned spot positions, we translated the coordinates recorded in the SPM plane to the isocenter plane by applying a projection factor to the SPM plane as described in Eqs. (1), (2).

(1) $$X_{\mathrm{iso}}=X_{\mathrm{SPM}}\times f_{projection\_x}$$

(2) $$Y_{\mathrm{iso}}=Y_{\mathrm{SPM}}\times f_{projection\_y}$$

where, $X_{\mathrm{iso}}$ and $Y_{\mathrm{iso}}$ are the coordinates defined at the isocenter plane, and $X_{\mathrm{SPM}}$ and $Y_{\mathrm{SPM}}$ are the coordinates recorded in the SPM plane. $f_{projection\_x}=1.696$ and $f_{projection\_y}=1.391$ are the projection factors for the x and y coordinates, respectively.

### 2.2 Statistical analysis of the errors

To characterize differences between planned and delivered values, histograms with Gaussian density estimates were used to visualize the distribution of MU deviations. For spot position deviations, errors in the x- and y-directions were evaluated separately, and the overall spot position deviations were assessed using the Euclidean distance, whose squared form follows a chi-square distribution under the assumption of independent coordinate errors.

Systematic error analyses were conducted for parameters whose mean deviations were persistently shifted from zero across multiple QA sessions. While histogram-based analyses were used to assess the overall distribution characteristics and zero-centered behavior of errors, systematic errors were defined based on persistent mean biases observed across repeated QA sessions, rather than from a single global distribution. Random errors, on the other hand, were defined as zero-centered fluctuations that vary unpredictably for each data point.

Statistical analyses were performed using SPSS v.27.0. (IBM Corp., Armonk, NY, USA) to evaluate differences in MU and spot-position errors across beam energies, treatment days, and treatment rooms using Levene’s test, Welch’s analysis of variance (ANOVA), and Games-Howell post hoc comparisons. Levene’s test was applied to evaluate the homogeneity of variance among groups at a significance level of 0.05 [17]. After determining the non-homogeneity of variance of errors for each of the categories, Welch’s ANOVA [18], [19] (p < 0.05) was performed to test for significant differences in errors in MU and spot position among the energies, treatment days, and treatment rooms. Welch’s ANOVA is an improvement of the commonly used one-way ANOVA and is used when groups have different variances. Welch’s ANOVA was then used to determine whether mean errors differed significantly among energies, treatment days, and rooms, with a significance level of 0.05. Post hoc analysis using the Games-Howell test identified which subgroups within energies, treatment days, and treatment rooms had significantly different errors in MU and spot position, without assuming homogeneity of variance, with a significance level of 0.05 [20].

### 2.3 Machine-learning-powered optimization

Predictive ML models were constructed to predict the delivered spot positions from the planned spot positions. To reduce information leakage and assess generalization to unseen future sessions, we used a temporal split, reserving the final month of data from each treatment room (16 files total) as the test set. Given the importance of accurately predicting both horizontal and vertical deviations separately, the target variables were the x- and y-coordinates of the delivered spot positions. Therefore, two separate predictive ML models were trained: one for predicting the x-coordinates and another for predicting the y-coordinates. To verify the independence between the horizontal and vertical steering systems, the correlation between x- and y-coordinates spot positions was evaluated using Pearson’s correlation coefficient. The training dataset included features consisting of each treatment room’s planned x- and y-coordinates, beam energy, and treatment date.

The ML models were implemented using the XGBRegressor from the xgboost library (v3.0.5), which provides a scikit-learn-compatible API. The scikit-learn package (v1.5.0) [21] was used for data preprocessing and model evaluation in Python (v3.10.16). The number of trees in the XGBRegressor was set to 500 for both the x- and y-coordinate models. A learning rate of 0.1 was used to control the contribution of each tree during the boosting process, and the models were trained using the squared error as the objective function.

The XGBRegressor [22] is an ensemble learning method that operates based on gradient boosting, building a series of decision trees sequentially during training. Each tree is constructed to minimize the gradient of a specified loss function, thereby reducing the overall model error iteratively. This approach allows XGBRegressor to capture complex, non-linear relationships between the features and the target variables and was chosen as the estimator for delivered spot positions.

After model training, feature importance was extracted from the trained models and quantified based on the average gain across all decision trees, representing the relative contribution of each feature to the prediction of spot delivery deviations.

### 2.4 Dose recalculation

To quantify the clinical impact of the predicted delivered spot positions on calculated dose distributions, voxel-wise dose distributions were recomputed for each test-set delivery log using an in-house analytical dose calculation engine commissioned for the Hitachi PROBEAT-V system. Each log file was converted into a DICOM ion plan reflecting the MU and predicted spot positions. We recalculated dose distributions from both the planned and predicted spot parameters using the same evaluation grid (2-mm isotropic) for fair comparison. For each dataset, statistics including mean, standard deviation, minimum, and maximum dose were extracted. Additionally, voxel-wise dose differences were computed, including the maximum absolute voxel-wise dose difference, defined as the largest absolute difference between the recomputed predicted and planned dose values at corresponding voxels.

### 2.5 Evaluation of ML models

The validation of the predictive ML models was conducted using planned position from the DICOM ion plan file as input to predict delivered position. The predicted delivered position was then compared with the recorded delivered position from the log file. The predictive models were assessed in terms of mean squared error (MSE), R^2^ score, and Euclidean distance. The MSE and R^2^ scores were calculated by $\mathrm{MSE}=\frac{100}{n}\sum_{t=1}^{n}{\left(A_{t}-P_{t}\right)}^{2}$ and $R^{2}score=1-\frac{\mathrm{SSR}}{\mathrm{SST}}$, where $A_{t}$ is the actual value and $P_{t}$ is the predicted value. The residual sum of squares (SSR) and the total sum of squares (SST) were defined as $\mathrm{SSR}=\sum_{t=1}^{n}{e_{t}}^{2}$, $\mathrm{SST}=\sum_{t=1}^{n}{\left(A_{t}-\overset{¯}{A}\right)}^{2}$, where $e_{t}$ is the residual value and $\overset{¯}{A}$ is the mean of actual values.

## 3 Results

### 3.1 Systematic error analysis

**Fig. 2:**
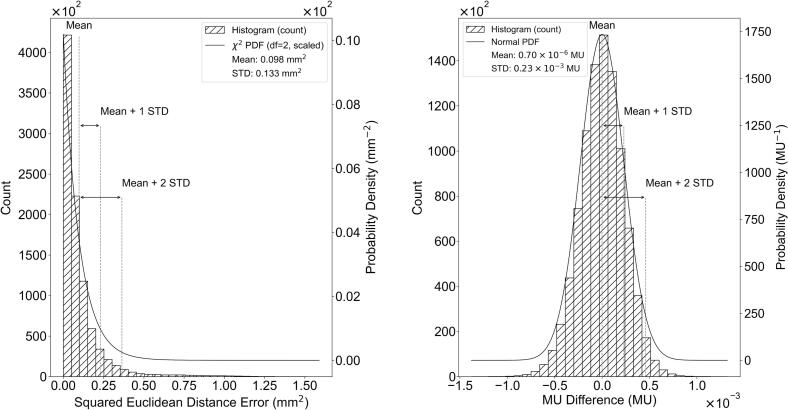
Distributions of differences between planned and delivered values for spot positions (A) and MU (B). Spot position deviations are quantified using the Euclidean distance, representing the overall magnitude of positional discrepancies combining the x- and y-direction errors. For MU, the difference distribution is shown with a Gaussian density estimate to illustrate its near-zero-centered behavior.

Fig. 2 presents the distributions of the differences between the planned and delivered values for MU and spot positions. For spot positions, deviations in the x- and y-directions were analyzed separately, as the ML models were trained independently for each coordinate. The x-coordinate errors followed an approximately normal distribution with a mean of 0.163 mm and a standard deviation of 0.199 mm, while the y-coordinate errors showed a mean of 0.096 mm and a standard deviation of 0.151 mm. Assuming independent and normally distributed errors in the x- and y-directions, the squared Euclidean distance follows a chi-square distribution with two degrees of freedom. Accordingly, the overall spot position deviation, expressed as the squared Euclidean distance, exhibited a mean value of 0.098 mm^2^, reflecting the overall magnitude of spot position variability.

For MU, the distribution was centered near zero, with a global mean of 0.70 × 10^−5^ MU, indicating negligible overall deviation. As no persistent mean shift was observed across repeated QA sessions, MU-related errors were excluded from further systematic error analyses.

**Fig. 3:**
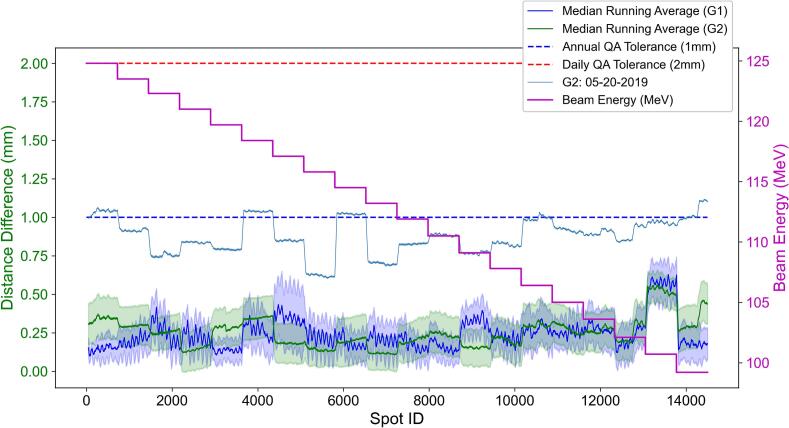
Systematic error analysis of the spot positions. The blue line and green line represent the running averages computed within each log file using a moving window for treatment rooms G1 and G2, respectively. The blue and green shaded bands indicate ±1 standard deviation across repeated QA sessions at each spot index. The purple line shows the beam energy (MeV) throughout the treatment. The blue dashed line and red dashed line indicate the annual QA tolerance and daily QA tolerance, respectively. The light blue line represents the running averages for the log files in which the values exceeded the annual QA tolerance. (For interpretation of the references to colour in this figure legend, the reader is referred to the web version of this article.)

Fig. 3 shows the systematic error of spot position errors over repeated QA sessions. While the distribution of spot position errors (Fig. 2) reflects overall variability, systematic errors were identified based on persistent mean deviations observed in the running averages computed within each log file using a moving window across multiple QA sessions. The moving-window size was set to 50 consecutive spots to reduce high-frequency spot-to-spot noise while preserving local systematic deviation patterns for visualization. For treatment room G2, the running average of spot position errors exhibited sustained deviations exceeding 1 mm on May 20th, surpassing the annual QA tolerance recommended by AAPM TG-224 [4]. On this day, a total of 2,669 spots exceeded the tolerance across multiple energy layers, indicating a session-specific systematic deviation rather than random fluctuations. In addition, Fig. 3 shows that treatment room G1 exhibited larger spot-to-spot fluctuations within the same energy layer compared to G2, suggesting increased short-range variability in spot position delivery.

### 3.2 Factors of errors

The detailed results of Levene’s test and Welch’s ANOVA evaluating differences in spot position errors across beam energies, treatment days, and treatment rooms are provided in Supplementary Table S1. Levene’s test indicated significant heterogeneity of variances across all categories (p < 0.001), supporting the use of Welch’s ANOVA for group comparisons. Welch’s ANOVA revealed statistically significant differences in spot position errors across energy levels, treatment days, and treatment rooms. Among these factors, beam energy showed the largest variability in spot position errors, followed by treatment day, while treatment room exhibited smaller but still statistically significant differences.

### 3.3 Performance of machine-learning-powered model

**Fig. 4:**
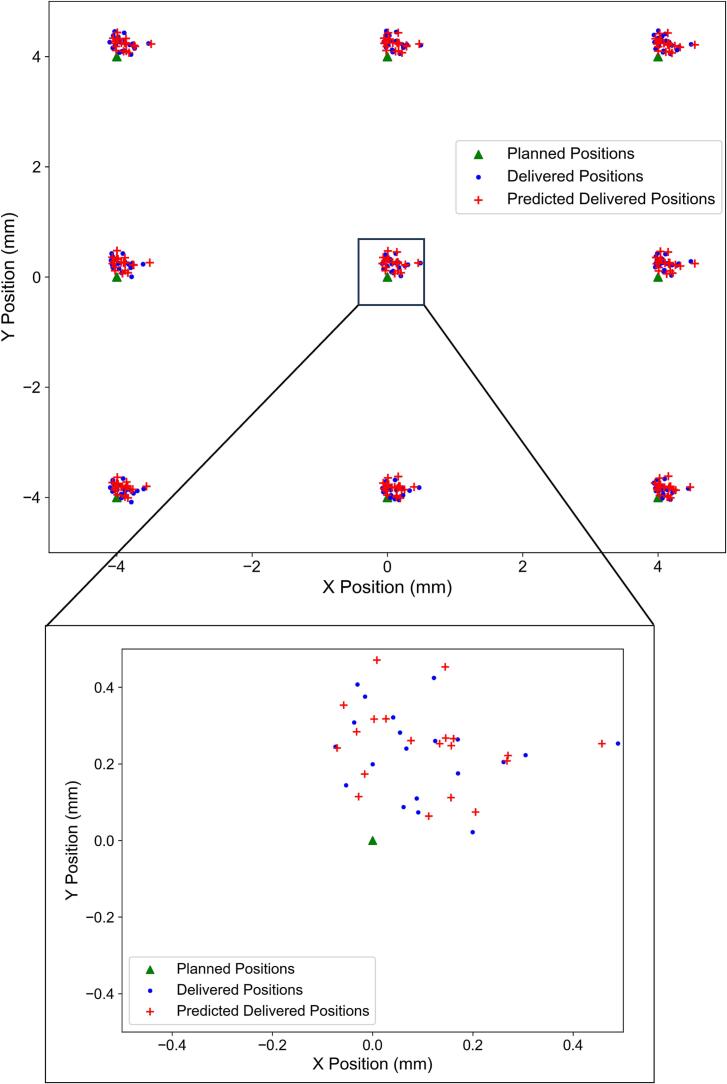
Comparison of planned, delivered, and predicted delivered spot positions for a single test-set session. The green triangles represent the planned positions, the blue rectangles represent the delivered positions, and the red plus signs represent the predicted delivered positions. The zoomed view shows the same session, focusing on a portion of the x- and y-axes, from −0.5 mm to 0.5 mm in both directions. (For interpretation of the references to colour in this figure legend, the reader is referred to the web version of this article.)

Predictive ML models were trained with a total number of 696,000 spots (48 files) and tested with a total number of 232,000 spots (16 files). Fig. 4 compares the predicted delivered spot positions with the actual delivered spot positions for both x- and y-coordinates, demonstrating the high predictive accuracy of the models. The correlation between x- and y-coordinate spot deviations was slightly negative (r = −0.086), indicating negligible cross-axis dependency between the two steering directions.

Table 1, Table 2 list the numerical performances of two ML models (for x- and y-coordinates) for predicting the delivered spot positions from the planned spot positions. Based on the results, overall ML models for the x-coordinates achieved a MSE of 0.021, while the model for the y-coordinates achieved a MSE of 0.003, with both models obtaining an R^2^ score of 0.999. Table 1, Table 2 further outline the model’s performance across different factors. Table 1 highlights the model performance across different treatment rooms and days, showing that the maximum Euclidean distance was 0.244 mm for G1 and 0.108 mm for G2. Table 2 presents the model performance across different beam energy ranges, showing that the mean Euclidean distance remained below 0.188 mm for all energy levels, indicating stable predictive performance across the investigated energy spectrum.

**Table 1:** Machine learning model performance in predicting delivered spot positions from planned spot positions across different treatment days.

TreatmentRoom	Date	x-coordinates		y-coordinates		Euclidean Distance (mm)
		MSE (mm^2^)	R^2^ score	MSE (mm^2^)	R^2^ score	
G1	6	0.008	0.999	0.001	0.999	0.079
	11	0.042	0.999	0.002	0.999	0.194
	14	0.045	0.999	0.002	0.999	0.199
	18	0.016	0.999	0.003	0.999	0.114
	21	0.025	0.999	0.003	0.999	0.150
	24	0.062	0.999	0.001	0.999	0.235
	25	0.053	0.999	0.001	0.999	0.216
	28	0.063	0.999	0.003	0.999	0.244

G2	6	0.003	0.999	0.005	0.999	0.082
	11	0.002	0.999	0.001	0.999	0.053
	14	0.002	0.999	0.003	0.999	0.067
	18	0.002	0.999	0.003	0.999	0.067
	21	0.006	0.999	0.002	0.999	0.083
	24	0.002	0.999	0.002	0.999	0.058
	25	0.002	0.999	0.010	0.999	0.107
	28	0.006	0.999	0.007	0.999	0.108

Overall		0.021	0.999	0.003	0.999	0.128

Abbreviations: MSE, mean squared error.

**Table 2:** Performance of machine learning models for predicting delivered spot positions from planned spot positions across different beam energy ranges.

Treatment Room	Beam Energy (MeV)	x-coordinates		y-coordinates		Mean Euclidean Distance (mm)
		MSE (mm^2^)	R^2^ score	MSE (mm^2^)	R^2^ score	
G1	90–109	0.043	0.999	0.002	0.999	0.188
	110–119	0.035	0.999	0.002	0.999	0.170
	120–129	0.040	0.999	0.002	0.999	0.180

G2	90–109	0.003	0.999	0.004	0.999	0.075
	110–119	0.004	0.999	0.004	0.999	0.081
	120–129	0.003	0.999	0.005	0.999	0.078

Overall mean		0.021	0.999	0.003	0.999	0.128

Abbreviations: MSE, mean squared error.

Feature importance analysis showed that the planned spot coordinates were the dominant predictors in both models. In the x-coordinates model, planned x-coordinates contributed most to the prediction (81.1%), followed by treatment room (18.4%) and beam energy (0.47%). In the y- coordinates model, planned y-coordinates showed the highest importance (82.5%), followed by treatment room (17.0%) and beam energy (0.64%).

### 3.4 Dose comparison results

To evaluate the dosimetric impact of the ML-based predicted spot positions, dose distributions were recomputed for each test set delivery log and compared against the original planned dose distributions. The mean dose values from the predicted spot-based calculations were in strong agreement with those from the planned spot-based calculations across all datasets. The average absolute difference in mean dose between the planned and predicted distributions was 2.340 × 10^−5^ Gy, with a standard deviation of 1.406 × 10^−5^ Gy.

Voxel-wise dose difference analysis showed that the maximum absolute voxel-wise dose difference was 1.796 Gy. This value corresponds to the largest dose difference observed at a single voxel between the predicted and planned dose distributions, indicating a localized worst-case discrepancy rather than an overall shift in dose. Across the test cases, the standard deviation of voxel-wise differences ranged from 0.038 to 0.108 Gy, reflecting the magnitude of local deviations at the voxel level.

## 4 Discussion

This feasibility study demonstrates that ML can accurately predict delivered spot positions in pencil beam scanning proton therapy with sub-millimeter accuracy. Our ML-powered tool focuses on comparing MU values and spot positions from the routine QA treatment plan with those recorded in delivery log files, and on predicting delivered spot positions based on plan data. We employed advanced ML algorithms to create a more efficient, accurate, and comprehensive method for identifying and analyzing discrepancies. Rather than focusing solely on numerical performance, our approach highlights how ML can be leveraged to detect subtle but clinically significant deviations in spot positions and MU values, which in many cases may not be readily apparent from conventional single-session QA analyses. This work contributes to the broader effort to improve the consistency and traceability of delivery-related data throughout the treatment workflow, complementing established QA practices that emphasize data integrity, such as checksum-like verification strategies used in treatment planning systems [23].

Several groups have demonstrated log-file-based QA for proton therapy systems showing that delivery logs can accurately reconstruct delivered dose distribution with high fidelity [9], [10], [11], [12]. In our approach, we predict delivered parameters based on plan data. The framework may provide an additional data-driven, predictive perspective on delivery behavior and may help identify deviations that require further review. Rather than replacing measurement-based QA, this framework is intended to complement existing QA approaches.

When designing the ML-based predictive model, we considered that the x- and y-coordinates of spot positions may be subject to different physical correction mechanisms and magnetic field influences. Accordingly, two independent predictive models were constructed separately for the x- and y-coordinates. The input features were selected based on domain knowledge and included clinically relevant factors that could affect delivery accuracy, such as beam energy, treatment date, and treatment room. Although both treatment rooms were matched within vendor acceptance criteria, residual differences in spot position variability were observed, with lower variability in G2 than in G1, potentially reflecting room-specific differences in beam optics. With this design, the trained models demonstrated high spatial prediction accuracy, with the predicted positions closely matching the actual delivered positions. Across the entire test set, the average Euclidean distance was 0.128 mm, which is below the 1 mm annual QA tolerance recommended by AAPM TG-224 [4]. This result indicates that the model is capable of accurately capturing subtle deviations that may occur during beam delivery.

In addition to spatial prediction accuracy, we assessed the dosimetric impact of using the ML-predicted spot positions by recomputing voxel-wise dose distributions on the test set and comparing them with the planned doses. The recalculated doses demonstrated strong agreement with the planned distributions, with an average absolute difference in mean dose as low as 2.340 × 10^−5^ Gy and a standard deviation of 1.406 × 10^−5^ Gy. Importantly, local voxel-wise differences remained within ±1.796 Gy. This value represents the largest local difference observed at a single voxel, rather than the overall dosimetric disagreement between the two dose distributions. This indicates that the maximum voxel-level discrepancies were confined to spatially localized regions and had negligible influence on the average absolute difference in mean dose. The narrow standard deviation range (0.038–0.108 Gy) across all test cases further supports the finding that such deviations were infrequent and spatially limited. These findings demonstrate that, despite localized variation, the ML-based QA framework achieves consistent and dosimetrically stable performance on unseen data, enabled by its high spatial prediction accuracy.

Despite the advantages and innovation mentioned, it is important to note that the accuracy of our ML models heavily depends on the quality and quantity of the training data. Our study was based on a single routine QA treatment plan with repeated delivery logs from our institution. Therefore, generalizability to broader plan types remains limited. Accordingly, extension of the current model to substantially different plan types should be supported by additional validation. Future validation will include multiple QA plans and real patient treatment plans to assess model robustness across clinical settings. The dataset used for this feasibility study was at a fixed gantry angle, which was not included as a feature in our models. Extension to other gantry angles is planned as the next phase of validation, which will assess whether angle-dependent corrections improve prediction accuracy and whether the model generalizes across the full range of clinical gantry positions. Furthermore, the energy range analyzed in this study was limited to a subset of energies sampled from routine QA measurements and does not span the full clinical energy range of the PROBEAT-V system. Extension to a broader energy spectrum will be addressed in future work to evaluate model performance under more clinically diverse beam conditions.

In addition, while our ML models achieved high predictive accuracy for spot position discrepancies, the model’s ability to support routine QA workflows between treatment fractions remains to be fully evaluated. The present study does not define a fixed retraining interval for clinical deployment. Instead, model updating should be guided by prospective performance monitoring using newly accumulated QA log data and performed when sustained drift or major system changes are observed. Log files are typically written at the end of each treatment session, allowing analysis post-treatment. ML predictions could flag concerning patterns for physicist review before the next fraction. The implementation of these models in a live clinical setting may present unforeseen challenges, such as integration with existing treatment planning and delivery systems, data processing speeds, and the feasibility of timely error flagging within routine QA workflows performed between treatment fractions. Therefore, clinical trials and iterative refinements will be required to adapt the models for practical use.

This feasibility study demonstrates that ML-based quality assurance can accurately predict delivered spot positions in pencil beam scanning proton therapy with sub-millimeter accuracy. The proposed models demonstrated consistent predictive performance, accurately detecting delivery discrepancies and improving system-level QA reliability. This framework may provide a foundation for future extensions toward error detection and prospective decision support between treatment fractions within routine QA workflows.

## CRediT authorship contribution statement

**Sang Kyun Yoo:** Writing – original draft, Visualization, Validation, Software, Methodology, Investigation, Formal analysis. **Sridhar Yaddanapudi:** Writing – review & editing, Project administration, Investigation, Data curation, Conceptualization. **Bo Lu:** Writing – review & editing, Resources, Investigation. **Ethan Stolen:** Writing – original draft, Visualization, Formal analysis. **Siddhant Sen:** Writing – original draft, Visualization, Formal analysis. **Byongsu Choi:** Writing – original draft, Visualization, Formal analysis. **Jin Sung Kim:** Writing – review & editing, Funding acquisition. **Keith Furutani:** Writing – review & editing. **Chris Beltran:** Writing – review & editing. **James J. Sohn:** Writing – review & editing, Supervision, Investigation, Conceptualization.

## Funding

This research was supported by a grant of the Korea Health Technology R&D Project through the Korea Health Industry Development Institute (KHIDI), funded by the Ministry of Health & Welfare, Republic of Korea (grant number: HI23C0730).

## Declaration of competing interest

The authors declare that they have no known competing financial interests or personal relationships that could have appeared to influence the work reported in this paper.

## Appendix A Supplementary data

Supplementary Data 1 Spot position errors by energy, day, and room.
