# Supplementary material for: A feasibility study on a machine-learning-based quality assurance tool for spot-scanning proton therapy using delivery log files and treatment plans
Source: Phys Imaging Radiat Oncol. 2026 Apr 30;39:100987. doi: 10.1016/j.phro.2026.100987 (PMC13200111; doi:10.1016/j.phro.2026.100987)
Supplement: Supplementary Data 1 — Spot position errors by energy, day, and room. [file mmc1.docx]

**Supplementary Table S1.** Statistical analysis of spot position errors across different energy levels, treatment days, and treatment rooms.

| Variables | Test type | Statistic | p-value |
| --- | --- | --- | --- |
| Beam Energy (MeV) | Levene’s Test |  |  |
|  | Based on Mean | 942.953 | < 0.001 |
|  | Welch’s ANOVA | 14,874.438 | < 0.001 |
| Treatment day | Levene’s Test |  |  |
|  | Based on Mean | 10,383.927 | < 0.001 |
|  | Welch’s ANOVA | 4,580.577 | < 0.001 |
| Treatment Room | Levene’s Test |  |  |
|  | Based on Mean | 5,044.012 | < 0.001 |
|  | Welch’s ANOVA | 497.199 | < 0.001 |

Abbreviations: $df$, degrees of freedom; ANOVA, analysis of variance
